# Mistreatment of university students most common during medical studies

**DOI:** 10.1186/1472-6920-5-36

**Published:** 2005-10-18

**Authors:** Arja Rautio, Vappu Sunnari, Matti Nuutinen, Marja Laitala

**Affiliations:** 1 Department of Pharmacology and Toxicology, University of Oulu, FIN-90014 University of Oulu, Finland; 2 Department of Educational Sciences and Teacher Education, University of Oulu, FIN-90014 University of Oulu, Finland; 3 Department of Paediatrics, University of Oulu, FIN-90014 University of Oulu, Finland

## Abstract

**Background:**

This study concerns the occurrence of various forms of mistreatment by staff and fellow students experienced by students in the Faculty of Medicine and the other four faculties of the University of Oulu, Finland.

**Methods:**

A questionnaire with 51 questions on various forms of physical and psychological mistreatment was distributed to 665 students (451 females) after lectures or examinations and filled in and returned. The results were analysed by gender and faculty. The differences between the males and females were assessed statistically using a test for the equality of two proportions. An exact two-sided P value was calculated using a mid-P approach to Fisher's exact test (the null hypothesis being that there is no difference between the two proportions).

**Results:**

About half of the students answering the questionnaire had experienced some form of mistreatment by staff during their university studies, most commonly humiliation and contempt (40%), negative or disparaging remarks (34%), yelling and shouting (23%), sexual harassment and other forms of gender-based mistreatment (17%) and tasks assigned as punishment (13%). The students in the Faculty of Medicine reported every form of mistreatment more commonly than those in the Faculties of Humanities, Education, Science and Technology. Experiences of mistreatment varied, but clear messages regarding its patterns were to be found in each faculty. Female students reported more instances of mistreatment than males and were more disturbed by them. Professors, lecturers and other staff in particular mistreated female students more than they mistreated males. About half of the respondents reported some form of mistreatment by their fellow students.

**Conclusion:**

Students in the Faculty of Medicine reported the greatest amount of mistreatment. If a faculty mistreats its students, its success in the main tasks of universities, research, teaching and learning, will be threatened. The results challenge university teachers, especially in faculties of medicine, to evaluate their ability to create a safe environment conducive to learning.

## Background

The main tasks of universities are research, teaching and learning. The teaching atmosphere during undergraduate studies is important not only for learning but also for building positive attitudes towards one's professional identity and towards life-long learning. Attitudes, positive or negative, adopted during university studies will have an impact on the values and behaviour of students in their future working lives.

Various forms of mistreatment have been reported to occur in a variety of workplaces, including schools [1], universities [2-5] and the police force [6]. Mistreatment is a problem on a personal level and on the organisational and societal levels as well. In some cases mistreatment can even lead to alcohol and drug abuse [2,7]. Exposure to mistreatment has a significant inverse correlation with both job satisfaction and psychological health and well being [8].

Mistreatment is perceived by undergraduate students in the United States as a major source of stress [9], and such perceptions and their consequences are more prevalent among medical students than either students or faculty staff believe [3,4,10,11]. More than a third of the students at medical school have considered dropping out, and one fourth report that they would have chosen a different profession had they known in advance about the extent of the mistreatment they would experience in American medical schools [12]. Corresponding results have been obtained in Finland [13]. Also, more generally, high proportions of students who experience mistreatment suffer measurable psychopathological consequences [3,7]. Perceived mistreatment has been found to be a major source of stress during medical internship [9], and especially when this is consistent and systematic, it may significantly impair mental health and well-being among both university students and employees and affect their overall satisfaction with their work [2,4,7].

Becher [14] found in the UK and US that mistreatment in the cultures of different university disciplines can vary. Disciplines and departments differ both at the level of epistemic issues and in the quality of social relations and atmospheres and their ways of controlling and punishing students. Becher thinks that it is the moral order that defines the basic beliefs, values, norms and aspirations prevailing in each disciplinary culture. This forms the background ethos for each discipline, determining what is regarded as normal and ordinary and what is impossible, imaginary or extraordinary.

### Objectives

A report from two medical schools in Finland in the early 1990s showed that three out of every four students had experienced some kind of mistreatment by classmates, teachers, hospital staff or patients during their education [11]. The present study was undertaken to evaluate the prevalence of physical and psychological mistreatment among students in all faculties of Oulu University, including the Faculty of Medicine. One special purpose was to see whether there were any differences in the treatment of students between the Faculty of Medicine and the other faculties and whether it would be meaningful to discuss the characteristics in terms of a moral order.

## Methods

### Study design

Permission to perform this survey was obtained from the rector of the university and from the chief administrator and chief academic officer of each faculty. The protocol was accepted by the university ethics committee. The work was carried out mainly within a 3-week period.

After briefly explaining the survey and its purpose to the students, we distributed the questionnaire forms (see Additional file 1) to them after a lecture or an examination and continued to be present while they filled them in. The students were not allowed to discuss the questionnaire, but were told that they were to give their own personal, honest answer to each question anonymously.

### Survey questionnaire

The questionnaire was modified from that used in 1991 to evaluate two medical schools in Finland [11], which had in turn been based on that of Sheehan et al. [12] and Baldwin et al. [15]. In order to keep the questionnaire valid and to be able to compare the present results with those obtained earlier using a similar questionnaire, we kept modification of the questionnaire to an absolute minimum. Since it had originally been used only among students in medical faculties, we modified it to be applicable to students in all faculties by changing few phrasings, e.g. we did not specifically ask about mistreatment by nurses.

The first 13 questions (out of the total of 51) covered the student's background, i.e. faculty, age, gender, native language, marital status, religion, years of study and curriculum, socio-economic status and level of education of the person's father and mother. These were followed by 36 structured and 2 open-ended questions (see Additional file 1) covering different types of physical and psychological mistreatment such as sexual harassment and discrimination, verbal and psychological mistreatment and physical threats (Table 1). Each staff group was listed separately under each question: "How often, if ever, have any of the following persons mistreated you (each type of mistreatment was asked separately)?" and the following options were given: "never", "rarely (1–2 times)", "sometimes (3–4 times)" and "often (5 times or more)". Each item also had space for a written answer and an opportunity to give an example of the mistreatment. If the respondent reported mistreatment, he/she also answered the question: "How much did this mistreatment bother you?" In addition to personal perceptions of mistreatment, we also attempted to evaluate its general occurrence in the university by asking: "How often does each type of mistreatment occur at your university?" The same options were given: never, rarely, sometimes and often.

**Table 1:** Topics addressed in the questionnaire (see Additional file 1).

Number of questions	**Topics addressed**
1–13	Student background
14–31	Mistreatment and harassment
32–35	Sexual harassment or mistreatment
36–39	Racial, ethnic, religious or age discrimination
40–42	Threats to fail or give a low grade
43–46	Negative or disparaging remarks on study performance
47–49	Sleep deprivation
50	Immoral, unethical or other unacceptable treatment during studies (open-ended question)
51	Other forms of mistreatment (open-ended question)

### Students

The main target groups were first and second-year students and those who had been studying for four years or more, to investigate the occurrence of mistreatment in relation to the number of years of study. Altogether 665 students participated in the survey, representing 7% of the total at the university. The proportion varied according to faculty, being 11.5% in the Humanities, 6.1% in Education, 6.6% in Science, 18.9% in Medicine and 3.5% in Technology. The sample size for each faculty was designed to be sufficiently large that no single student or teacher could be identified in the analyses. The students who had been studying for more than three years in the Faculties of Education and Technology were doing their practical training period outside the university and could not be reached. Only a few students refused to fill in the questionnaire, and those who returned it had answered all the questions. The exact figures according to faculty, gender and study year, and the proportion (%) of female students in each faculty and among the respondents are given in Tables 2 and 3. The median number of years of study was two for the males and almost three for the females, and the median age was roughly the same for both sexes, between 22 and 23 years. All the participants were Finnish, and 90% of them were members of the Evangelical-Lutheran church. The female students were more often married (43%) than the male ones (34%).

**Table 2:** Percentages of female students in the faculties and among the students participating in the survey (n = 662).

Faculties	Percentage of women	
	In the faculty %	In the survey %
Humanities	75.6	77.8
Education	77.2	88.0
Medicine	67.4	80.2
Science	50.5	75.5
Technology	14.8	7.3

**Table 3:** Numbers of students participating in the survey, by faculty and study year (n = 634; 18 females and 10 males did not report the study year).

	**Students by years of study **Number										**Total **Number (%)	
Year	1		2		3		4		>4			
Sex*	F	M	F	M	F	M	F	M	F	M	F	M

Faculties:												
Humanities	23	3	16	8	17	7	15	9	55	9	126 (19.9)	36 (10.4)
Education	12	0	22	2	10	5	14	0	8	2	66 (10.4)	9 (1.4)
Medicine	1	0	35	9	16	5	23	5	39	9	114 (18.0)	28 (6.2)
Science	19	1	29	17	34	9	13	9	25	3	120 (18.9)	39 (6.2)
Technology	0	11	4	50	2	11	0	10	1	7	7 (1.1)	89(14.0)

Total	55	15	106	86	79	37	65	33	128	30	433 (68.3)	201 (31.7)

*F = female, M = male

### Statistical procedures

The data were analysed using SPSS (Statistical Package for Social Sciences, version 7.0) and the differences between the males and females were assessed using a test for the equality of two proportions [16] in the Arcus Quickstat Biomedical software (Research Solutions). An exact two-sided P value was calculated using a mid-P approach to Fisher's exact test (the null hypothesis being that there is no difference between the two proportions) [16].

## Results

### Mistreatment by staff

**Figure 1:**
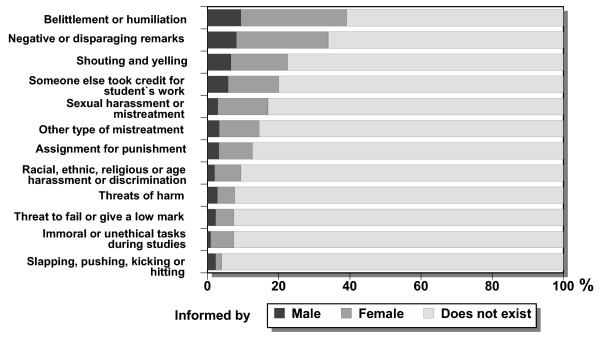
Percentages (%) of female and male students who reported given types of mistreatment by staff (n = 647 – 652).

Our results showed that mistreatment is common in the university, since 40% of the men and 55% of the women had experienced some mistreatment by staff or faculty members during their university studies (Table 4). Females more commonly reported mistreatment than males (p < 0.0005), and were more disturbed by it. Twenty-one percent of students reported at least one instance of mistreatment, and 12.6% reported having experienced four or more different types of mistreatment (Table 4). The most common form was belittlement and humiliation (40%) (Figure 1), followed by negative or disparaging remarks about the respondent's academic performance (34%), yelling and shouting (23%), sexual harassment and other forms of gender-based mistreatment (17%) and tasks assigned as punishment (13%). Research fellows and senior research fellows and lecturers were most often reported as the sources of this mistreatment. (Table 5). Professors, lecturers and other (non-academic) staff mistreated female students significantly more frequently than males (Table 5).

**Table 4:** Total numbers of episodes of mistreatment by staff (range 0–10) and fellow students (range 0–8) reported by students during their university studies.

**Frequency**	**Staff**			**Fellow students**		
	Male (N = 193) %	Female (N = 378) %	All (N = 571) %	Male (N = 205) %	Female (N = 426) %	All (N = 631) %

Never	60.1	44.7	49.9	54.1	48.8	50.6
Once	21.2	20.9	21.0	24.9	26.3	25.8
2–3 times	9.3	20.1	16.5	14.2	19.7	17.9
4–5 times	5.1	7.7	6.9	3.9	4.5	4.3
6 or more times	4.3	6.6	5.7	2.9	0.7	1.4

The data were obtained by counting how many types of mistreatment a single student reported having experienced. We recognised ten types of mistreatment by staff (questions 14,17,20,23, 26,29,32,36,40,43) and eight types of mistreatment by fellow students (questions 14,17,23,26, 29,32,36,43).

**Table 5:** Frequency (%) of all types of mistreatment by given categories of staff experienced by students.

**Staff categories**	**Male **%	**Female **%	**All **%
Professors	12.4	18.6*	16.6
Associate professors	13.4	13.7	13.6
Research/senior research fellows	26.2	28.6	27.7
Lecturers	16.9	33.3**	27.9
Other staff	8.7	15.5*	13.2

Differences between female and male students, *p < 0.05; **p < 0.0001.

**Figure 2:**
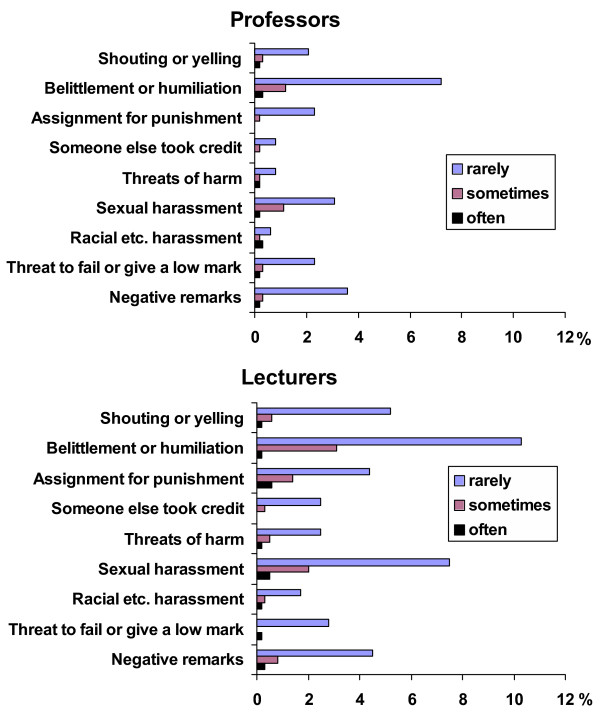
Occurrence and frequency of different types of mistreatment from professors and lecturers as reported by students. Rarely (1–2 times), sometimes (3–4) and often (5 or more times).

Belittlement and humiliation were the most common forms of student mistreatment for every staff group. The second most common among the professors was sexual harassment or gender-based mistreatment, together with negative or disparaging remarks. Among the lecturers it was sexual harassment or gender-based mistreatment together with assignments given as a punishment (Figure 2). Research fellows and senior research fellows were reported to shout and yell at students and to assign tasks as punishment. Shouting and yelling were the second most common form of mistreatment by other groups of staff.

### Mistreatment by fellow students

51.2% of the female students and 45.9% of the males reported having experienced mistreatment from fellow students at least once (Table 4). 24.5% of the females and 19.2% of the males reported contempt and humiliation, and derogatory remarks concerning the career chosen by the informant were common, as also was students taking credit for someone else's work (Table 6). The results showed that the students did not appreciate the fields of study pursued in other faculties, this being especially evident in the answers given by the students of the humanities and technology concerning each other's fields of study.

**Table 6:** Occurrence (%) of mistreatment by staff and fellow students during the 2^nd ^year and during or after the 4^th ^year, as reported by male and female students.

	**2nd year %**				**≥4th year %**				**All %**			
	*Staff*		*Fellow student*		*Staff*		*Fellow student*		*Staff*		*Fellow student*	

Number of answer (M = male, F = female)	M 84 – 86	F 102 – 104	M 85 – 86	F 102 – 104	M 61 – 62	F 184 – 189	M 62	F 187 – 191	M 208 – 211	F 433 – 446	M 208 – 211	F 437 – 451
Shouting or yelling	7.0	11.5	11.6	9.6	14.5	15.3	8.1	18.8	8.2	12.6	13.0	12.8
Belittlement or humiliation	10.5	23.1	15.1	24.0	26.2	28.9	29.0	31.7	15.5	26.3	19.2	24.5
Assignment for punishment	7.0	9.7	-	-	12.9	12.2	-	-	10.1	11.7	-	-
Someone else took credit for student's work	2.0	3.8	11.8	17.3	4.8	6.3	21.0	20.1	3.9	4.1	15.9	18.5
Threats to harm	3.5	2.9	3.5	0	3.2	6.3	9.7	2.7	3.4	5.7	6.7	2.0
Slapping, pushing etc.	0	0	8.1	1.0	1.6	0	14.5	2.6	0.5	0.2	8.7	2.5
Gender-based harassment or mistreatment	3.5	10.6	7.0	7.7	8.1	23.2	4.8	9.0	4.8	15.1	7.7	6.1
Racial, ethnic harassment or discrimination etc.	2.3	4.9	3.5	10.7	4.8	3.7	9.7	6.4	2.4	3.0	5.8	8.2
Threat to fail or give a low mark	2.3	3.9	-	-	9.8	9.1	-	-	5.3	6.2	-	-
Negative remarks	5.8	8.8	21.2	25.5	4.9	10.3	17.7	25.7	5.3	8.5	20.3	23.3

### Mistreatment in relation to years of study

The reported occurrence of mistreatment both by staff and fellow students increased with the number of years of study (Table 6). This was especially true of sexual harassment or gender-based mistreatment and threats to fail a student or give a low mark, which were reported 2–4 times as often during or after the 4^th ^year as in the 2^nd ^year both by males and females (Table 6). The same tendency was also observed in mistreatment by fellow students. Female students who had been studying for 4 years or more reported shouting and yelling to be twice as common as those who had been studying for 1 or 2 years (Table 6). Men reported the largest increase in belittlement and humiliation and in some one else taking credit for their work. There was no change in the reported occurrence of sexual harassment by fellow students over the years.

### Sexual harassment and other forms of gender-based mistreatment

**Figure 3:**
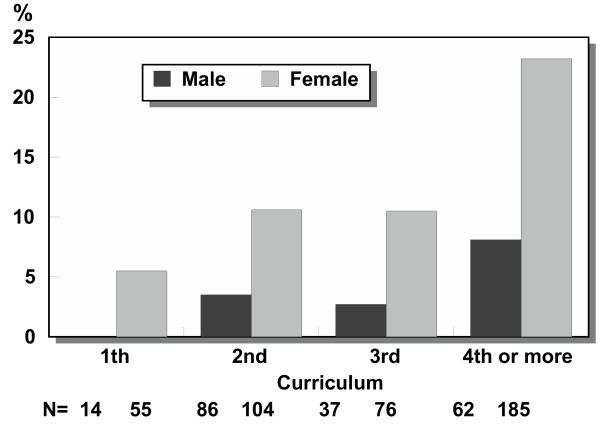
Sexual harassment or mistreatment by staff (%) as reported by male and female students, by years of study (N = number of students).

The female students reported gender-based mistreatment significantly more commonly than the males (p < 0.0001) and the frequency of this increased with the duration of studying (Figure 3). 21% of the female students and 10% of the males had either personally experienced or observed some form of sexual harassment or gender-based mistreatment during their studies. The occurrences of different forms of this on the part of teachers or other staff, as reported by female and male students, are given in Table 7. The most common types were derogatory remarks (sexist slurs), affecting 11.5% of the female students and 3.4% of the males, while 9.0% of the female students and 2.9% of the males had experienced gender-based discrimination (favouritism). Equal percentages of men and women (3.4%) reported having experienced sexual approaches (advances). The faculty staff mistreated female students more often than male ones (p = 0.0002), but mistreatment by fellow students was equally common among both. Sexual harassment or gender-based mistreatment was reported most often by the female students in the Faculty of Medicine (28.4%) and the Faculty of Humanities (24.2%), and the lowest figures reported by women were in the Faculty of Sciences (10.5%) (Table 8). 24.1% of the male respondents in the Faculty of Medicine reported sexual harassment or other forms of gender-based mistreatment. Of the categories of staff, lecturers were most often reported as sources of sexual harassment or discrimination (Figure 2).

**Table 7:** Reported occurrence (%) of different types of gender-based harassment or mistreatment by staff, by gender of the respondent.

**Type of sexual harassment or mistreatment**	**Males **(N = 211) %	**Females **(N = 451) %	**All **(N = 662) %
Denied opportunities	1.9	3.7	3.2
Exchange of rewards for sexual favours	1.0	0	0.3
Advances	3.4	3.4	3.4
Sexist slurs	3.4	11.5	8.9
Sexist teaching material	1.0	2.0	1.7
Malicious gossip	2.9	1.6	2.0
Favouritism	2.9	9.0	7.0
Poor evaluations	2.4	3.8	3.4

**Table 8:** Occurrence of different types of mistreatment by staff, by respondent's gender and faculty.

Faculties	**Medicine**		**Humanities**		**Education**		**Technology**		**Science**	
	Male N = 28–29	Female N = 106–111	Male N = 36–38	Female N = 130–134	Male N = 11	Female N = 67–68	Male N = 90–91	Female N = 8	Male N = 40	Female N = 123–124
	% (N)	% (N)	% (N)	% (N)	(N)^a)^	% (N)	% (N)	(N)^a)^	%(N)	% (N)

Shouting or yelling	14.3 (4)	21.6 (24)	2.6 (1)*	21.6 (29)*	(3)	11.8 (8)	12.1 (11)	(2)	10.0 (4)	11.3 (14)
Belittlement or humiliation	32.1 (9)	43.2 (48)	21.1 (8)	32.6 (43)	(1)	35.3 (24)	13.2 (12)	(3)	30.0 (12)	29.3 (36)
Assignment for punishment	27.6 (8)	22.5 (25)	5.3 (2)	7.5 (10)	(1)	19.1 (13)	4.4 (4)	(2)	15.0 (6)	10.5 (13)
Threats of harm	6.9 (2)	7.3 (8)	5.3 (2)	5.3 (7)	0	7.4 (5)	4.4 (4)**	(1)**	7.5 (3)	5.6 (7)
Threat to fail or give a low mark	17.2 (5)	6.5 (7)	10.5 (4)	7.6 (10)	(1)	5.9 (4)	4.4 (4)	0	5.0 (2)	5.7 (7)
Negative or disparaging remarks	13.8 (4)	18.9 (20)	13.9 (5)	10.0 (13)	(1)	9.0 (6)	3.3 (3)	0	7.5 (3)	10.6 (13)
Gender-based harassment or mistreatment	24.1 (7)	28.4 (31)	13.2 (5)	24.2 (32)	(3)	22.1 (15)	3.3 (3)***	(3)***	7.5 (3)	10.5 (13)

^a) ^No percentage is given because of the small number of answers.

Statistical significances of differences between females and males within the same faculty: * p < 0.01, ** p < 0.05, *** p < 0.0001

### Immoral, unethical and other unacceptable treatment during studies

There were two open questions concerning immoral and unethical treatment or other unacceptable treatment. 47 students reported that they had to do something immoral or unethical during their studies (Table 9), the female students reporting this more frequently than the males (9.6% vs. 3.0%; p = 0.0031). The largest student group reporting immoral and unethical treatment was female students in the Faculty of Medicine (17.1%). The medical students wrote in their open answers that they had to treat patients who were "too sick" or dying, and that this caused them anxiety. The students of science quoted environmental problems with chemicals. The only form of religious discrimination, reported by one student, was favouritism in the attitudes of teachers who were members of a small local religious group towards students belonging to the same group.

**Table 9:** Occurrence (%) of immoral, unethical and other unacceptable treatment during studies, as reported by male and female students.

	**Immoral, unethical treatment**		**Other unacceptable treatment**	
Faculty	Male (N = 203) %	Female (N = 429) %	Male (N = 203) %	Female (N = 425) %
Medicine	3.6	17.1	10.3	15.7
Humanities	0	3.1	16.2	17.3
Education	0	5.9	0	14.7
Technology	2.3	14.3	10.2	14.3
Science	7.7	11.6	7.9	17.4

Total	3.0*	9.6*	10.3**	16.5**

*Differences between male and female students, *p = 0.0031; **p = 0.041

The last question concerned all other types of mistreatment during university studies. This was answered by 91 students, with the female students reporting such things more than the males (p = 0.041). Other unacceptable treatment was reported most often by females in the faculties of Medicine (15.7%), Humanities (17.3%) and Science (17.4%). Most of these comments were concerned with teaching skills, poor teacher-student relations, the atmosphere in a department, nasty behaviour by office secretaries and practical training in teaching. The answers to these open-ended questions reported disagreements within and between faculties, e.g. between dentists and medical students or doctors and nurses, or between students in the Faculty of Technology and either the Faculty of Humanities or the Faculty of Education. Students in the Faculty of Education also reported that teachers gave assignments as a means of punishment, that they threatened students, and that they were lacking in punctuality and were in the habit of cancelling their lectures at the last moment.

### Overall estimation of mistreatment in the university

**Figure 4:**
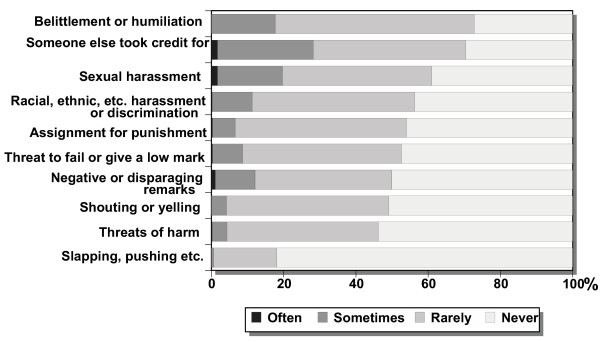
Overall perceptions of different types of mistreatment as reported by students (n = 629 – 650) when asked in the form: "How often does each type of mistreatment occur at your university?".

The frequencies of personally experienced mistreatment (Figure 1) were lower than the overall perceptions of mistreatment during university studies (Figure 4). Female students reported more mistreatment in the university overall than did the male students (Figure 4).

### Differences between the faculties

The results suggest that the faculties have their own "typical" modes of mistreatment (Table 8). Belittlement or humiliation were especially often reported by males in the Faculties of Medicine (32.1%) and Science (30.0%), whereas the respective figure in Technology was 13.2%. Among females the highest figures were in Medicine (43.2%), followed by Education (35.3%). Negative or disparaging remarks were reported most often by females (18.9%) and males (13.8%) in Medicine, in contrast to only 3.3% of males (3 out of 90) in Technology. Shouting and yelling was rarely reported by males in the Humanities (2.6%), whereas the females in the same faculty reported this 9 times more often (21.6%). Threats to fail a student or to give a low grade were reported most often by male students of Medicine (17.2%). Assignments given as punishment were evident in the Faculties of Medicine (males 27.6%; females 22.5%) and Education (females 19.1%). The gender that was in the minority in a faculty regularly reported mistreatment more often than the majority gender, e.g. female students in the Faculty of Technology.

## Discussion

Mistreatment appeared to be very common, since about half of the students had experienced some form of mistreatment by staff or faculty members and every fifth student reported at least one instance of mistreatment. This can sometimes be explained by racial, ethnic or social discrimination [17,18], but that is not the case here, since the social, ethnic and racial backgrounds of our students were very homogeneous. Some students will evidently be taken as victims of extensive mistreatment, as can be seen from the fact that 25 female students (6.6%) and eight male students (4.3%) reported having experienced as many as six or more different types of mistreatment. These high figures demand particular attention. The sense of victimization is a complex issue related to negative identification, the sociological and psychological environment and personality [19]. The reporting of any form of abuse or mistreatment is to some extent subjective and depends on the personality and psycho-social structure of the respondent, and thus vulnerable to over reporting. It is difficult to generalize on results of this kind, but in the light of figures reported also by other authors [3,5,7,15,20] mistreatment of medical students seems to be very common.

Also, about half of the students reported some form of mistreatment by their fellow students. Belittlement, humiliation and negative remarks were astonishingly common. "Inappropriate comments" by fellow-students were also the major category of harassment identified by White [5]. In our material students seemed not to appreciate each other's fields of study, and the present atmosphere in certain disciplines evidently does not support collaboration. It is important that the teachers should not express attitudes of their own that belittle other faculties or disciplines.

The occurrence of mistreatment generally increased with the number of years of study, as also reported earlier [5,10,21,22]. A significant difference has been reported, for example, between the percentages of second-year and third-year medical students quoting any experience of mistreatment (37% vs. 76%) [21]. Furthermore, in accordance with some other studies [3,5,23,24], the women were treated worse than men and were more seriously disturbed by the treatment. It was surprising that medical students reported mistreatment most often in the present survey, even though Uhari et al. [11] had also reported high rates of negative experiences, up to 70%, among students in two Finnish faculties of medicine in the early 1990s and a frequency of 37% for sex-based mistreatment. Very similar rates was reported by White [5] at an Australian medical school, where 37.9% of the students reported some form of sexual harassment (male 24.6%; female 47.9%). The female respondents accounted for 80% of all the events reported, and often reported the same form of harassment more than once.

Twenty-one percent of the female students and 10% of the male students had either personally experienced or observed some form of gender-based mistreatment or discrimination during their studies, the frequency being 2.5-fold among fourth- year students or over by comparison with first-year students. These figures are in accordance with those of White [5], who reported that sexual harassment at medical school was highly significantly (P < 0.001) more common during the clinical years 4–6 than during the pre-clinical years 1–3. Sexual harassment and other sex-based forms of mistreatment experienced especially by women can be ways of trying to marginalize women in university settings [25]. They can have this effect in practice whether or not they are intended as such, because it is not exhilarating to seek a career in a field where you may be harassed sexually and marginalized [26]. High figures for sexual and gender-related harassment have been reported by Larsson [3] in Sweden, for example.

The high figures for mistreatment by lecturers and teachers without tenure may point to signs of frustration. Teaching is no longer held in high regard in universities by comparison with research work, which is appreciated and respected far more. Recent changes in the working environment have also resulted in insecurity, which may be reflected in the treatment of students by such categories of staff. These issues nevertheless cannot provide a full explanation for the mistreatment that students encounter while studying at university.

It is possible that the use of aversive methods to make students learn and behave better has been passed down from teacher to learner, i.e. there is a "transgenerational legacy" that leads to future mistreatment of others by those who themselves have been mistreated. Also, to add to the picture, it is possible that the attitudes, forms of behaviour and values that characterise study cultures and atmospheres in faculties may be connected with discipline-based or professional socialisation, or different moral orders, to use the terminology of Becher [14] discussed at the beginning of this paper. The students' experiences of mistreatment in different faculties gave some messages regarding attitudes and hidden assumptions that can be interpreted as being connected with the kind of discipline-based moral order that Becher discusses [14]. This type of discipline-based mistreatment was most evident in medicine and education, fields in which academic socialisation can be greatly influenced by the norms of socialisation that are traditionally emphasised in the professions for which the students are being educated. These norms can exercise a highly covert influence and form tacit but fundamental assumptions and prejudices [14].

The forms of mistreatment discussed by the students of medicine were very often connected with the position of the student. In a faculty of medicine, as in a hospital, one's position (doctor, nurse, patient, student) seems to be central, and is based on a clear hierarchy within the system. Sexual harassment can be conceptualized as an institutionally sanctioned display of the power that the harasser believes he/she possesses in relation to the victim [5]. This power is often thought to be obtained and derived either from gender or from formal status in the workplace, but it may also play a role in situations where there is no apparent power over the victim, such as a patient harassing a doctor. In such cases the victim may hold the power but the harasser exercises a contra-power and may cause harassment in spite of the apparent formal power imbalance. This may also be true in the case of harassment by a fellow student.

The position of a student in the hierarchy is not so clear as that of a professional, especially when practising in a hospital. The position of an individual in a hospital is often connected with the work done with patients: e.g. who is in a position to decide about injections, operations etc, whose task is it to perform each action, who is allowed to have a voice in each situation and who is not. In that kind of setting it is easy to understand that the atmosphere can be characterised by a strong measure of position-based control and the punishment and mistreatment connected with it, i.e. the use of power. These issues thus characterise the moral order of the study culture. The treatment of students of education – especially by their teachers – points to different areas of control and punishment from those that apply to medicine, but which also relate to the use of power, since teachers control their pupils' use of time, the nature of the tasks they have to do, etc. Correspondingly, the special topics emphasised by the trainee teachers were the control maintained over their time and attendance. The only comment on an obligation to be present in lectures or exercises in spite of being ill was made by a student of teacher education.

The topic of sexual harassment can also be approached through the concept of moral order. Another study conducted by us [27] gives more detailed information on the situation in the Faculty of Technology, noting that although the respondents indicated that they had not experienced sexual harassment, some other students claimed to have left that faculty because of the sexually harassing atmosphere they had experienced. It is thus possible to think that the moral order of the Faculty of Technology, or of some of its departments, may include the idea that as a female student you must abide a sexually harassing atmosphere if you wish to study there.

In both the Faculties of Medicine and in that of Education the female students reported mistreatment more often than the males, as if the order were stricter for them. The question of treatment is especially important in these faculties, for several reasons. One is that most of the students in these faculties are female and mistreatment seems to disturb them more than males. On the other hand, the nature of this treatment is an especially important question in the professional areas concerned, as it is a question of how teachers treat the pupils for whom they are responsible and how doctors treat their patients. These aspects need further study.

## Conclusion

For most people the vision of universities is that they are peaceful sanctuaries protected from the "real world", where students are taught high-level academic skills, read, write and do research. It is also thought that equity, dignity, respect and justice are emphasized in these environments. But, as earlier [17,18,28,29], the present survey shows that this is not true. Students are not treated equally in our universities.

The main tasks of a university – research, teaching and learning – are seriously threatened if members of staff mistreat their students, and this is especially true where women are concerned. The teaching atmosphere during one's studies is important not only for learning but also for building up a positive professional identity. The attitudes adopted during university studies, positive or negative, will have an impact on graduates' values and behaviour in their future working life. And of course, a university hires its researchers and teachers of tomorrow from today's students. Therefore, it is very important to prevent mistreatment from being transmitted to the next generation, in order to increase professionalism in medicine [30], as in other disciplines. This may be achieved by various methods, such as role playing [30] and educational strategies [31].

Our findings emphasize the need to develop and maintain a good, impartial and supportive atmosphere within medical studies and training in order to develop the personality traits needed to practise as a doctor. Serious discussion is obviously needed on the behaviour and habits of teaching staff. Indeed, some degree of educational intervention is needed in all faculties of a university. Issues of the hidden moral order should also be included in these discussions. Several interesting perspectives for educational interventions have been provided by White [31] and Heru [30], for example, and by B Sandler and R Shoop [32].

## Competing interests

The author(s) declare that they have no competing interests.

## Authors' contributions

AR participated in the planning, execution, analysing and writing the manuscript. VS participated in analysing and writing the manuscript. MN participated in the planning, execution, analysing and writing the manuscript. ML participated in the planning, execution, analysing and writing the manuscript. All authors read and approved the final manuscript.

## Pre-publication history

The pre-publication history for this paper can be accessed here:



## Supplementary Material

Additional File 1 Questionnaire. Click here for file
