# Supplementary material for: Mistreatment of university students most common during medical studies
Source: BMC Med Educ. 2005 Oct 18;5:36. doi: 10.1186/1472-6920-5-36 (PMC1285362; doi:10.1186/1472-6920-5-36)
Supplement: Additional File 1 — Questionnaire. [file 1472-6920-5-36-S1.doc]

Appendix 1.

### Questionnaire

At the beginning (questions 1–13) we asked about the student’s background (faculty, age, sex, native language, marital status, religion, years of study and curriculum and the socio-economic status and level of education of the person’s father and mother). We then went on to ask the following:

Please answer all the questions: circle the correct response and give examples in the space provided.

14. How often, if ever, have any of the following persons shouted or yelled at you?

|  | Never | Rarely  (1–2 times) | Sometimes  (3–4 times) | Often  (5 or more times) | Examples |
| --- | --- | --- | --- | --- | --- |
| a. Fellow students | 0 | 1 | 2 | 3 |  |
| b. Professors | 0 | 1 | 2 | 3 |  |
| c. Assistant professors | 0 | 1 | 2 | 3 |  |
| d. Assistants | 0 | 1 | 2 | 3 |  |
| e. Lecturers | 0 | 1 | 2 | 3 |  |
| f. Other staff | 0 | 1 | 2 | 3 |  |

15. How much did the shouting or yelling bother you?

Does not apply not at all a little a lot

0 1 2 3

16. How often does this shouting or yelling occur at your university?

Never Rarely Sometimes Often

1 2 3 4

17. How often, if ever, have any of the following persons belittled or humiliated you?

|  | Never | Rarely  (1–2 times) | Sometimes  (3–4 times) | Often  (5 or more times) | Examples |
| --- | --- | --- | --- | --- | --- |
| a. Fellow students | 0 | 1 | 2 | 3 |  |
| b. Professors | 0 | 1 | 2 | 3 |  |
| c. Assistant professors | 0 | 1 | 2 | 3 |  |
| d. Assistants | 0 | 1 | 2 | 3 |  |
| e. Lecturers | 0 | 1 | 2 | 3 |  |
| f. Other staff | 0 | 1 | 2 | 3 |  |

18. How much did the belittlement or humiliation bother you?

Does not apply not at all a little a lot

0 1 2 3

19. How often does this belittlement or humiliation occur at your university?

Never Rarely Sometimes Often

1 2 3 4

20. How often, if ever, have any of the following persons assigned you tasks, work, or rotation responsibilities as a punishment rather than for educational purposes?

|  | Never | Rarely  (1–2 times) | Sometimes  (3–4 times) | Often  (5 or more times) | Examples |
| --- | --- | --- | --- | --- | --- |
| a. Professors | 0 | 1 | 2 | 3 |  |
| b. Assistant professors | 0 | 1 | 2 | 3 |  |
| c. Assistants | 0 | 1 | 2 | 3 |  |
| d. Lecturers | 0 | 1 | 2 | 3 |  |
| e. Other staff | 0 | 1 | 2 | 3 |  |

21. How much did this punishment bother you?

Does not apply not at all a little a lot

0 1 2 3

22. How often does this practice occur at your university?

Never Rarely Sometimes Often

1 2 3 4

23. How often, if ever, have any of the following persons taken credit for work you have done (e.g. papers, projects, clinical work, or research)?

|  | Never | Rarely  (1–2 times) | Sometimes  (3–4 times) | Often  (5 or more times) | Examples |
| --- | --- | --- | --- | --- | --- |
| a. Fellow students | 0 | 1 | 2 | 3 |  |
| b. Professors | 0 | 1 | 2 | 3 |  |
| c. Assistant professors | 0 | 1 | 2 | 3 |  |
| d. Assistants | 0 | 1 | 2 | 3 |  |
| e. Lecturers | 0 | 1 | 2 | 3 |  |
| f. Other staff | 0 | 1 | 2 | 3 |  |

24. How much did the fact that someone else took credit for your work bother you?

Does not apply not at all a little a lot

0 1 2 3

25. How often does this happen at your university?

Never Rarely Sometimes Often

1 2 3 4

26. How often, if ever, have any of the following persons threatened to harm you?

|  | Never | Rarely  (1–2 times) | Sometimes  (3–4 times) | Often  (5 or more times) | Examples |
| --- | --- | --- | --- | --- | --- |
| a. Fellow students | 0 | 1 | 2 | 3 |  |
| b. Professors | 0 | 1 | 2 | 3 |  |
| c. Assistant professors | 0 | 1 | 2 | 3 |  |
| d. Assistants | 0 | 1 | 2 | 3 |  |
| e. Lecturers | 0 | 1 | 2 | 3 |  |
| f. Other staff | 0 | 1 | 2 | 3 |  |

27. How much did the threats bother you?

Does not apply not at all a little a lot

0 1 2 3

28. How often do such threats occur at your university?

Never Rarely Sometimes Often

1 2 3 4

29. How often, if ever, have any of the following persons slapped, pushed, kick or hit you?

|  | Never | Rarely  (1–2 times) | Sometimes  (3–4 times) | Often  (5 or more times) | Examples |
| --- | --- | --- | --- | --- | --- |
| a. Fellow students | 0 | 1 | 2 | 3 |  |
| b. Professors | 0 | 1 | 2 | 3 |  |
| c. Assistant professors | 0 | 1 | 2 | 3 |  |
| d. Assistants | 0 | 1 | 2 | 3 |  |
| e. Lecturers | 0 | 1 | 2 | 3 |  |
| f. Other staff | 0 | 1 | 2 | 3 |  |

30. How much did this bother you?

Does not apply not at all a little a lot

0 1 2 3

31. How often does this occur at your university?

Never Rarely Sometimes Often

1 2 3 4

32. How often, if ever, have any of the following persons subjected you to sexual harassment or discrimination (e.g. favouritism, advances, slurs, sexist teaching material)?

|  | Never | Rarely  (1–2 times) | Sometimes  (3–4 times) | Often  (5 or more times) | Examples |
| --- | --- | --- | --- | --- | --- |
| a. Fellow students | 0 | 1 | 2 | 3 |  |
| b. Professors | 0 | 1 | 2 | 3 |  |
| c. Assistant professors | 0 | 1 | 2 | 3 |  |
| d. Assistants | 0 | 1 | 2 | 3 |  |
| e. Lecturers | 0 | 1 | 2 | 3 |  |
| f. Other staff | 0 | 1 | 2 | 3 |  |

33. How much did the sexual harassment or discrimination bother you?

Does not apply not at all a little a lot

0 1 2 3

34. How often does sexual harassment or discrimination occur at your university?

Never Rarely Sometimes Often

1 2 3 4

35. If you have been subjected to sexual harassment or discrimination, what form did it take? (Check all that apply)

a. Denied opportunities

b. Exchange of rewards for sexual favours

c. Sexual advances

d. Sexist slurs

e. Sexist teaching material

f. Malicious gossip

g. Favouritism

h. Poor evaluations

36. How often, if ever, have any of the following persons subjected you to racial, religious, age or ethnic discrimination (e.g. prejudice, slurs, favouritism)?

|  | Never | Rarely  (1–2 times) | Sometimes  (3–4 times) | Often  (5 or more times) | Examples |
| --- | --- | --- | --- | --- | --- |
| a. Fellow students | 0 | 1 | 2 | 3 |  |
| b. Professors | 0 | 1 | 2 | 3 |  |
| c. Assistant professors | 0 | 1 | 2 | 3 |  |
| d. Assistants | 0 | 1 | 2 | 3 |  |
| e. Lecturers | 0 | 1 | 2 | 3 |  |
| f. Other staff | 0 | 1 | 2 | 3 |  |

37. How much did the discrimination bother you?

Does not apply not at all a little a lot

0 1 2 3

38. How often does such discrimination occur at your university?

Never Rarely Sometimes Often

1 2 3 4

39. If you have been subjected to racial, religious, age or ethnic discrimination, what form did it take? (Check all that apply)

a. Denied opportunities

b. Racial or ethnic slurs

c. Racist teaching material

d. Malicious gossip

e. Favouritism

f. Poor evaluations

40. How often, if ever, have any of the following persons threatened to fail you unfairly or give you an unjustifiably bad grade?

|  | Never | Rarely  (1–2 times) | Sometimes  (3–4 times) | Often  (5 or more times) | Examples |
| --- | --- | --- | --- | --- | --- |
| a. Professors | 0 | 1 | 2 | 3 |  |
| b. Assistant professors | 0 | 1 | 2 | 3 |  |
| c. Assistants | 0 | 1 | 2 | 3 |  |
| d. Lecturers | 0 | 1 | 2 | 3 |  |
| e. Other, who?  ___________________ | 0 | 1 | 2 | 3 |  |

41. How much did such threats bother you?

Does not apply not at all a little a lot

0 1 2 3

42. How often do threats of this kind occur at you university?

Never Rarely Sometimes Often

1 2 3 4

43. How often, if ever, have any of the following persons made negative or disparaging remarks about your future profession or career in science?

|  | Never | Rarely  (1–2 times) | Sometimes  (3–4 times) | Often  (5 or more times) | Examples |
| --- | --- | --- | --- | --- | --- |
| a. Fellow students | 0 | 1 | 2 | 3 |  |
| b. Professors | 0 | 1 | 2 | 3 |  |
| c. Assistant professors | 0 | 1 | 2 | 3 |  |
| d. Assistants | 0 | 1 | 2 | 3 |  |
| e. Lecturers | 0 | 1 | 2 | 3 |  |
| f. Other staff | 0 | 1 | 2 | 3 |  |
| g. Family members | 0 | 1 | 2 | 3 |  |

44. Did the negative or disparaging remarks bother you?

Does not apply not at all a little a lot

0 1 2 3

45. How often do you hear negative or disparaging remarks about your future profession or career in science?

Never Rarely Sometimes Often

1 2 3 4

46. How often do you hear negative or disparaging remarks about studying in general at your university?

Never Rarely Sometimes Often

1 2 3 4

47. How often have you experienced periods of prolonged sleep deprivation during your university studies?

Never 0

A few times a quarter 1

Weekly 2

More than once a week 3

Almost daily 4

48. How often have you experienced periods of prolonged sleep deprivation during your university studies because of the following:

Never Rarely Occasionally Often

a. Studying to keep up with course requirements 0 1 2 3

b. Preparing for course exams 0 1 2 3

c. Preparing for finishing your studies/finding work 0 1 2 3

49. Please indicate the degree to which you agree or disagree with each of the following statements on the scale provided.

Scale: Strongly agree = 1

Agree = 2

Disagree = 3

Strongly disagree = 4

_____ a. Sleep deprivation is an unfortunate but necessary part of training.

_____ b. The time I went without sleep was worth it because of what I learned.

_____ c. Sleep deprivation has little direct value in training me to become a good professional.

_____ d. I feel that doing without sleep has sometimes impaired my capacity to study.

_____ e. When I have to do without sleep, I am moodier and more short-tempered.

_____ f. During periods of sleep deprivation, I got into more conflicts with the professional staff.

50.Have you ever been required to do anything during your university studies that you thought was immoral, unethical, or otherwise personally unacceptable ?

□ yes

□ no

If yes, please explain:

51. Have you experienced any other forms of mistreatment during your university time?

□ yes

□ no

If yes, please describe one or more examples in as much detail as possible, and indicate how often they have occurred:
